# Emergency medical service utilization among acute ischemic stroke patients in Beijing: An observational study

**DOI:** 10.3389/fneur.2022.969947

**Published:** 2022-09-06

**Authors:** Kexin Ding, Hui Chen, Yong Wang, Hongmei Liu, Bayier Ceceke, Wei Zhang, Ling Geng, Guifang Deng, Tao Sun, Wenzhong Zhang, Yiqun Wu

**Affiliations:** ^1^Department of Epidemiology and Biostatistics, School of Public Health, Peking University, Beijing, China; ^2^Department of Internet Management and Quality Control, Beijing Emergency Medical Center, Beijing, China; ^3^Beijing Emergency Medical Center, Beijing, China

**Keywords:** emergency medical services, acute ischemic stroke, risk factors, urban-rural disparities, prehospital delay

## Abstract

**Objective:**

To investigate emergency medical service (EMS) utilization and its associated factors in patients with acute ischemic stroke (AIS), and further explore the urban-rural differences.

**Methods:**

Medical records for AIS in all emergency departments in Beijing were obtained from the Beijing Emergency Care Database from January 2018 to December 2021. EMS utilization was described and factors associated with EMS use were examined by multivariable logistic regression models with the generalized estimating equations. Results were compared between urban and rural districts.

**Results:**

A total of 24,296 AIS patients were included in the analysis, and 11,190 (46.1%) were transported to hospitals by EMS. The percentage of EMS usage in urban areas was significantly higher than that in rural areas (53.6 vs. 34.4%, *P* < 0.001). From 2018 to 2021, EMS utilization was on the increase (*P*-value for trend <0.001) with a higher average annual growth rate in rural areas (12.6%) than in urban (6.4%). Factors associated with EMS utilization were age (OR: 1.20 per 10-year increase, 95% CI: 1.17–1.23), NIHSS scores, off-hour arrival (OR: 1.32, 95% CI: 1.23–1.37), treatment in tertiary hospitals (OR: 1.75, 95% CI: 1.60–1.92), and possessing comorbidities such as coronary artery disease (OR: 1.15, 95% CI: 1.17–1.24), atrial fibrillation (OR: 1.56, 95% CI: 1.41–1.73), prior stroke (OR: 0.84, 95% CI: 0.78-0.90) or dyslipidemia (OR: 0.78, 95% CI: 0.71–0.85).

**Conclusion:**

This study demonstrated an inadequate use of EMS among AIS patients in Beijing, especially in rural areas, and revealed several associated factors. Enhanced education programs and EMS accessibility are necessary particularly for high-risk individuals and regions.

## Introduction

Acute ischemic stroke (AIS) is one of the leading causes of mortality and disability in China and around the world (1–4). Early diagnosis and timely treatment are paramount to achieve maximal functional recovery (5). Emergency medical services (EMS) utilization among AIS has been proven to significantly shorten prehospital delay and enhance prenotification of the receiving hospital (5), and rapid thrombolytic treatment was observed subsequently (6–8). Many studies have shown that the main challenges in stroke management in China are pre-hospital delays and a relatively low thrombolysis rate, while the main benefits of EMS are quicker triage and higher rates of thrombolysis (9, 10). However, the rate of EMS utilization (12.5%) is currently low among Chinese AIS patients in a national study in 2018 (7). Among other regional studies in China, the prevalence of EMS utilization shows a difference due to various distributions of medical resources and patient awareness in space and time (11–13). To improve the management of AIS acute care in China it is important to monitor the use of pre-hospital EMS and respond to changing patterns. Equally considerable is that such adequate care should be delivered in both urban and rural regions. Yet the use and determinants of health and emergency services likely differ between rural and urban regions, on account of different healthcare infrastructure, characteristics, and preferences of patients (14).

A program targeted at streamlining AIS care processes in prehospital and stroke facilities has been implemented in Beijing since January 2018 (15). Using the data acquired by this program, we aimed to describe the pattern of EMS utilization, identify associated factors with EMS activation, and compare them between urban and rural areas. The results may provide valuable information to achieve appropriate EMS planning and develop effective interventions to reduce delays and improve outcomes for AIS patients.

## Methods

### Data source and study population

**Figure 1:**
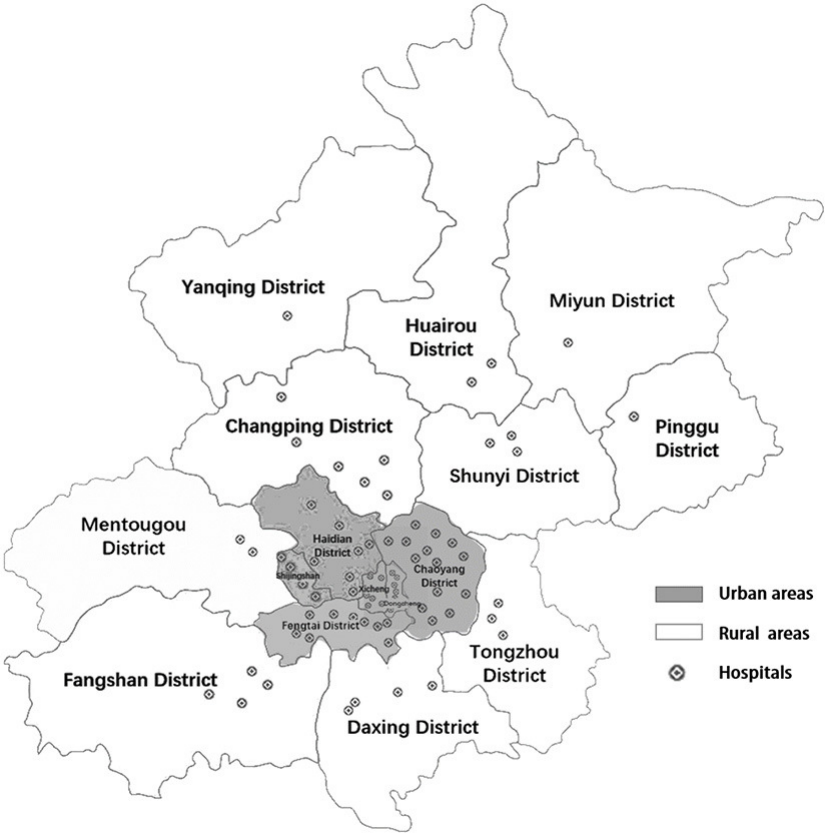
Seventy four hospitals in the First Aid Treatment Map for Stroke (FATMS) in Beijing.

An observational study was conducted based on historical data from Jan. 2018 to Dec. 2021. Data were obtained from the Beijing Emergency Care Database (16). The database keeps records of medical information for several conditions (acute stroke, heart attack, injury, and gynecological diseases) in the EMS system and emergency departments in secondary and tertiary hospitals in Beijing. This prospective registry was established in January 2018 and has been updated in real-time since then (15). For acute stroke, patients from all the 74 hospitals eligible for AIS acute care, included in the First Aid Treatment Map for Stroke (FATMS) published by Beijing Health and Family Planning Commission (Figure 1; Supplementary Table 1), were all recorded by a standard platform. Paramedics, physicians, and nurses in all eligible centers were trained to streamline the AIS emergency management processes by using this platform. For patients transported by the ambulance, once the paramedics identified a suspected IS patients, the nearest qualified hospital will be prenotified to clear a fast pathway for the coming patients. During transportation, paramedics initiated the patient's records on the platform. For patients who arrivied the hospitals by themselevs, nurses in the emergency departments would notify the AIS acure care team and start the records on the platform directively (15). Records of patients who arrived at the hospital within 4.5 h of onset were kept in the database. The main variables included demographic information, comorbidities, ways of transportation, types of treatment, diagnosis at discharge, hospital information, and so on. Patients with the primary diagnosis of AIS from January 2018 to December 2021 were included in this study. After excluding in-hospital stroke and transferred patients, a total of 24,296 AIS patients were included in the analysis.

### Outcome measure and key variables

Patients who were transported to hospitals by ambulance were identified as using EMS. The main outcome of interest was the percentage of EMS use in AIS patients. Other key variables included in the analysis were demographic features (age and sex), stroke severity, time of hospital arrival, comorbidity, the level of receiving hospitals, and district (urban or rural areas). Stroke severity was determined by the National Institutes of Health Stroke Scale (NIHSS) score and stratified as <6, 6 to 15, and >15. Time of hospital arrival was defined as on-hour arrival (8 am to 6 pm) or off-hour arrival (6 pm to 8 am). Comorbidity was concerned with the presence of vascular-related diseases, including hypertension, dyslipidemia, diabetes mellitus, prior stroke, heart failure (HF), atrial fibrillation, coronary artery disease, carotid stenosis (CS), and peripheral vascular disease (PVD) (7). The receiving hospitals were classified as secondary hospitals or tertiary hospitals, according to the Beijing Municipal Health Commission. The district variable (urban or rural areas) depended on where the patient lives according to the classification of the National Bureau of Statistics of China. Six districts of Beijing were classified as urban (including Dongcheng district, Xicheng district, Chaoyang district, Haidian district, Shijingshan district and Fengtai district), while the remaining ten districts were classified as rural (Figure 1). The differences in the accessibility and effectiveness of the urban and rural health services were striking according to previous studies (17).

### Statistical analysis

Characteristics of AIS patients were first described by frequencies and percentages for categorical variables, mean and standard deviation (SD) for continuous variables with normal distribution, and median and interquartile range (IQR) for continuous variables with skewed distribution. Characteristics of patients between urban and urban areas were then compared by the chi-square test, Student's *t*-test, or Wilcoxon test. Factors associated with EMS utilization were further examined by fitting multivariable logistic regression models with the generalized estimating equations to account for within-hospital clustering. Odds ratios (ORs) and 95% confidence intervals (CIs) were reported after adjusting for individual and hospital characteristics, including age, sex, baseline NIHSS group, comorbidities, time of hospital arrival, and level of receiving hospital. Besides, the calendar year was added as an adjustment variable in the sensitivity analysis. All analyses were conducted by different districts. Statistical significance was set at a *P*-value of 0.05. All analyses were conducted in R (v.4.1.0).

## Results

### Characteristics of AIS patients

A total of 24,296 AIS patients were analyzed with a mean age of 65.6 (SD: 12.8) years and 68.4% of males. The average baseline NIHSS score was 7.9 (SD: 6.9). Over three-quarters of patients had one or more vascular-related comorbidities. Two-fifths of patients arrived at the hospital from 6 pm to 8 am and a quarter of patients arrived on weekend. The characteristics of the patients were shown in Table 1. There were 14,766 (60.8%) and 9,530 (39.2%) patients treated in urban and rural areas, respectively. Patients in urban areas were older, had higher NIHSS scores, were more likely to present face drooping or slurred speech, arrive at hospitals on off-hour, possess comorbidities, and be treated in tertiary hospitals (Table 1).

**Table 1:** Characteristics of AIS patients.

	**Overall** **(*N* = 24,296)**	**Urban** **(*N* = 14,766)**	**Rural** **(*N* = 9,530)**	***P-*value**
Age, years, mean (sd)	65.6 (12.8)	66.6 (13.1)	64.1 (12.1)	<0.001
Male, *n* (%)	16,620 (68.4)	10,059 (68.1)	6,561 (68.8)	0.242
NIHSS score				<0.001
0–5	11,587 (47.7)	6,702 (45.4)	4,885 (51.3)	
6–16	98,74 (40.6)	6,221 (42.1)	3,653 (38.3)	
>16	28,35 (11.7)	1,843 (12.5)	992 (10.4)	
**Comorbidity**, ***n*** **(%)**				
Hypertension	14,240 (58.6)	8,556 (57.9)	5,684 (59.6)	0.009
Diabetes mellitus	6,111 (25.2)	3,952 (26.8)	2,159 (22.7)	<0.001
Prior stroke	6,036 (24.8)	3,810 (25.8)	2,226 (23.4)	<0.001
Coronary artery disease	4,505 (18.5)	2,882 (19.5)	1,623 (17.0)	<0.001
Dyslipidemia	2,917 (12.0)	1,955 (13.2)	962 (10.1)	<0.001
Atrial fibrillation	2,672 (11.0)	1,874 (12.7)	798 (8.4)	<0.001
HF/CS/PVD^*^	330 (13.6)	189 (12.8)	141 (14.8)	0.209
Off-hour arrival, *n* (%)	9,950 (41.0)	6,209 (42.0)	3,741 (39.3)	<0.001
Weekend arrival, *n* (%)	6,706 (27.6)	4,055 (27.5)	2,651 (27.8)	0.555
Level of hospitals, *n* (%)				<0.001
Secondary hospitals	2,752 (11.3)	1,039 (7.0)	1,713 (18.0)	
Tertiary hospitals	21,544 (88.7)	13,727 (93.0)	7,817 (82.0)	

* HF, heart failure; CS, carotid stenosis; PVD, peripheral vascular disease.

### EMS utilization

**Figure 2:**
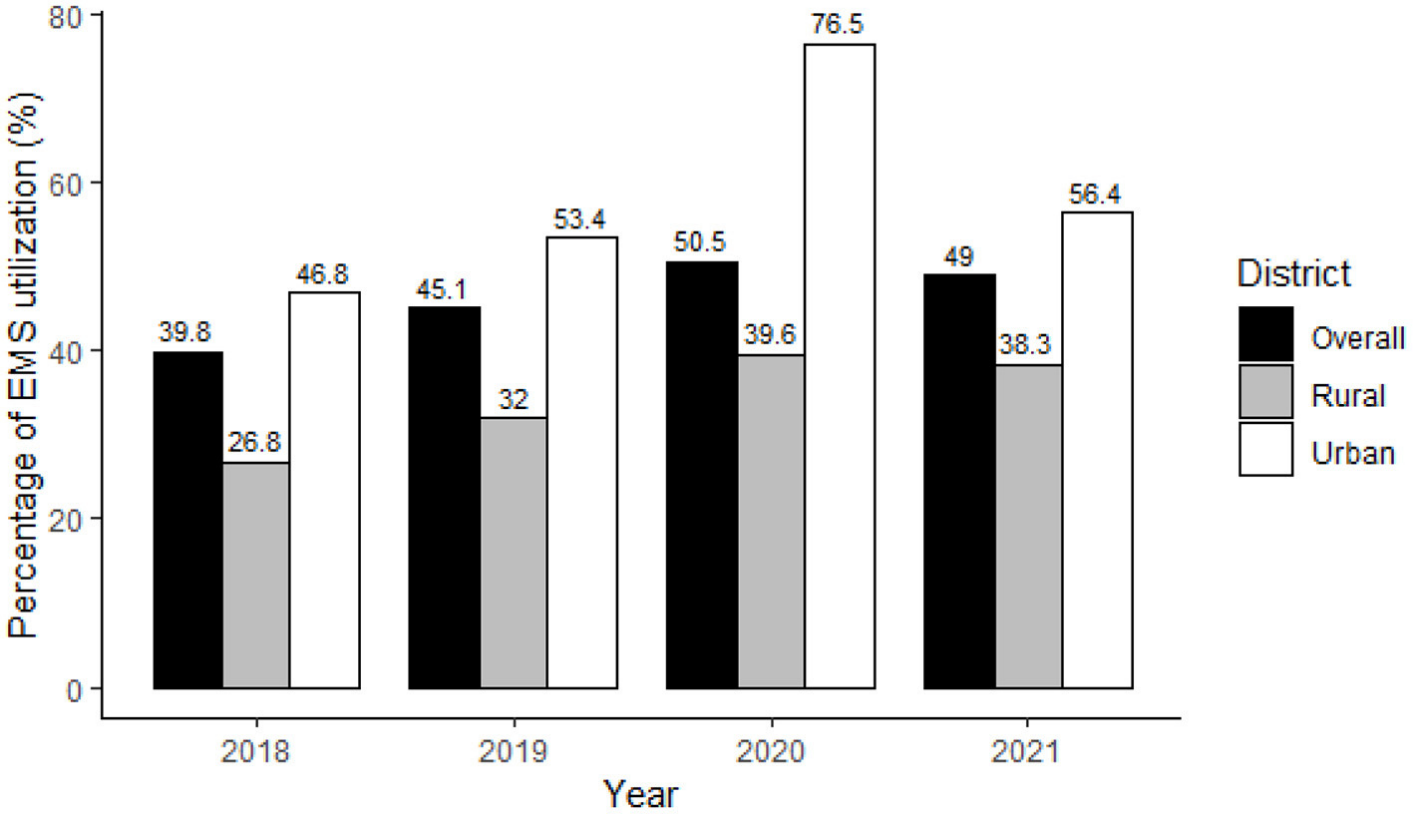
Percentage of EMS utilization in AIS patients in Beijing, 2018–2021.

Among all AIS patients, 11,190 (46.1%) were transported to hospitals by EMS, and the percentage of EMS usage in urban areas was significantly higher than in rural areas (53.6 vs. 34.4%, *P* < 0.001). From 2018 to 2021, EMS utilization was on the increase in both areas (both *P*-values for trend <0.001), with a higher rate in urban areas each year (Figure 2). The average annual growth rate in rural areas was higher than in urban areas (12.6 vs. 6.4%, *P* < 0.001). The higher percentages of EMS usage in urban areas were shown in patients with different characteristics, except for those treated in secondary hospitals (Table 2).

**Table 2:** Percentage of EMS utilization in AIS patients with different characteristics.

	**Urban**, ***n*** **(%)**		**Rural**, ***n*** **(%)**		***P-*value**
	**EMS**	**non-EMS**	**EMS**	**non-EMS**	
**Age**					
18–59	1,992 (45.0)	2,431 (55.0)	922 (27.6)	2,423 (72.4)	<0.001
60–74	3,136 (52.0)	2,893 (48.0)	1,414 (33.4)	2,824 (66.6)	<0.001
75+	2,782 (64.5)	1,532 (35.5)	944 (48.5)	1,003 (51.5)	<0.001
**Sex**					
Male	5,174 (51.4)	4,885 (48.6)	2,217 (33.8)	4,344 (66.2)	<0.001
Female	2,736 (58.1)	1,971 (41.9)	1,063 (35.8)	1,906 (64.2)	<0.001
**NIHSS group**					
0–5	2,283 (34.1)	4,419 (65.9)	943 (19.3)	3,942 (80.7)	<0.001
6–16	4,050 (65.1)	2,171 (34.9)	1,637 (44.8)	2,016 (55.2)	<0.001
>16	1,577 (85.6)	266 (14.4)	700 (70.6)	292 (29.4)	<0.001
**Comorbidity**					
Hypertension	4,638 (54.2)	3,918 (45.8)	1,987 (35.0)	3,697 (65.0)	<0.001
Diabetes mellitus	2,086 (52.8)	1,866 (47.2)	724 (33.5)	1,435 (66.5)	<0.001
Prior Stroke	1,912 (50.2)	1,898 (49.8)	782 (35.1)	1,444 (64.9)	<0.001
Coronary artery disease	1,756 (60.9)	1,126 (39.1)	674 (41.5)	949 (58.5)	<0.001
Atrial fibrillation	1,376 (73.4)	498 (26.6)	458 (57.4)	340 (42.6)	<0.001
Dyslipidemia	875 (44.8)	1,080 (55.2)	276 (28.7)	686 (71.3)	<0.001
HF/CS/PVD^*^	127 (67.2)	62 (32.8)	40 (28.4)	101 (71.6)	<0.001
Off-hour arrival	3,631 (58.5)	2,578 (41.5)	1,363 (36.4)	2,378 (63.6)	<0.001
Weekend arrival	2,230 (55.0)	1,825 (45.0)	932 (35.2)	1,719 (64.8)	<0.001
**Level of hospital**					
Secondary hospital	342 (32.9)	697 (67.1)	571 (33.3)	1,142 (66.7)	0.854
Tertiary hospital	7,568 (55.1)	6,159 (44.9)	2,709 (34.7)	5,108 (65.3)	<0.001

* HF, heart failure; CS, carotid stenosis; PVD, peripheral vascular disease.

Compared with patients in the non-EMS groups, patients in the EMS group were older, more likely to be females, more severe, more likely to possess comorbidities, and more likely to be received intravenous thrombolysis therapy with door-to-needle time ≤60 min (Supplementary Table 2).

### Factors associated with EMS utilization

Factors associated with the use of EMS were older age (OR: 1.20 per 10-year increase, 95% CI: 1.17–1.23), higher baseline NIHSS scores, off-hour arrival (OR: 1.30, 95% CI: 1.23–1.37), treatment in tertiary hospitals (OR: 1.75, 95% CI: 1.60–1.92), and possessing comorbidities such as coronary artery disease (OR: 1.15, 95% CI: 1.07–1.24) and atrial fibrillation (OR: 1.56, 95% CI: 1.41–1.73). Factors associated with not use of EMS were possessing comorbidity of prior stroke (OR: 0.84, 95% CI: 0.78–0.90) or dyslipidemia (OR: 0.78, 95% CI: 0.71–0.85).

When further stratified by district, factors associated with EMS utilization were similar in rural and urban regions except for sex, off-hour arrival, possessing HF/CS/CVD, and treatment in tertiary hospitals (Table 3). The association between sex and EMS usage was only observed in urban areas but not in rural areas. Possessing HF/CS/CVD was significantly associated with the tendency of EMS utilization only in the rural area but not in the urban area, while the association between EMS utilization and treatment in tertiary hospitals was only significant in the urban area. The association between EMS utilization and off-hour arrival was stronger in the urban area than in the rural area. The results were similar when added calendar year as an adjustment variable in the sensitivity analysis (Supplementary Table 3).

**Table 3:** Factors associated with EMS utilization^*^.

	**Total**		**Urban**		**Rural**		**Interaction**
	**OR (95%CI)**	***P-*value**	**OR (95%CI)**	***P-*value**	**OR (95%CI)**	***P-*value**	***P-*value**
Age per 10 years	1.20 (1.17, 1.23)	<0.001	1.18 (1.15, 1.22)	<0.001	1.19 (1.14, 1.24)	<0.001	0.650
**Sex**							
Female	ref		ref		ref		
Male	1.06 (1.00, 1.13)	0.059	1.00 (0.92, 1.08)	0.974	1.12 (1.01, 1.24)	0.025	0.076
**NIHSS group**							
0–5	ref		ref		ref		
6–16	3.19 (3.01, 3.39)	<0.001	3.14 (2.90, 3.41)	<0.001	3.26 (2.95, 3.60)	<0.001	0.622
>16	7.98 (7.18, 8.88)	<0.001	8.46 (7.30, 9.81)	<0.001	7.95 (6.77, 9.33)	<0.001	0.306
**Comorbidity**							
Hypertension	1.02 (0.96, 1.08)	0.522	1.04 (0.96, 1.12)	0.377	1.07 (0.97, 1.18)	0.154	0.889
Diabetes mellitus	1.03 (0.96, 1.10)	0.462	1.00 (0.92, 1.09)	0.956	0.98 (0.88, 1.10)	0.768	0.979
Prior stroke	0.84 (0.78, 0.90)	<0.001	0.79 (0.72, 0.86)	<0.001	0.88 (0.79, 0.98)	0.025	0.125
Coronary artery disease	1.15 (1.07, 1.24)	<0.001	1.17 (1.06, 1.29)	0.002	1.14 (1.00, 1.29)	0.044	0.716
Atrial fibrillation	1.56 (1.41, 1.73)	<0.001	1.49 (1.31, 1.69)	<0.001	1.50 (1.26, 1.77)	<0.001	0.785
Dyslipidemia	0.78 (0.71, 0.85)	<0.001	0.74 (0.66, 0.83)	<0.001	0.77 (0.65, 0.91)	0.002	0.582
HF/CS/PVD	0.91 (0.71, 1.17)	0.463	1.26 (0.89, 1.79)	0.192	0.65 (0.44, 0.98)	0.038	0.012
Off-hour arrival	1.30 (1.23, 1.37)	<0.001	1.37 (1.27, 1.47)	<0.001	1.16 (1.05, 1.27)	0.002	0.003
Weekend arrival	1.03 (0.97, 1.10)	0.321	1.04 (0.96, 1.13)	0.297	1.02 (0.92, 1.14)	0.649	0.832
**Level of hospital**							
Secondary hospital	ref		ref		ref		
Tertiary hospital	1.75 (1.60, 1.92)	<0.001	2.04 (1.76, 2.37)	<0.001	1.07 (0.95, 1.21)	0.286	<0.001

* HF, heart failure; CS, carotid stenosis; PVD, peripheral vascular disease. OR and 95% CI were obtained by multivariable logistic regression models with the generalized estimating equations to account for within-hospital clustering. Variables included in multivariable models were age, sex, baseline NIHSS group, comorbidities, time of hospital arrival, and level of receiving hospital.

## Discussion

Timely delivery of reperfusion therapies is paramount for the neurological recovery of AIS patients. EMS utilization is reportedly the most significant and consistent factor associated with reduced prehospital delays among AIS patients. To approve the probabilities of timely treatment, activation of EMS after a stroke attack is recommended by recent guidelines (18). Since January 2018, a program aimed at streamlining the AIS care processes in prehospital has been implemented in Beijing including promoting the use of ambulances. Based on a 4-year observation, we investigate EMS utilization and its associated factors in patients with acute ischemic stroke (AIS) and further explored the urban-rural difference.

According to the results, less than half (46.1%) of AIS patients were transported *via* ambulance in Beijing. Previous studies revealed the EMS utilization rates in other provinces and cities in China were only 9.1–32.9% (11–13), with a national reported rate of 12.5% in 2018 (7). The percentage of patients who use EMS in Beijing was higher than in other places in China. Even though, the percentage of EMS usage in this study was much lower than the reported 63.7% in the United States (6), 78.8% in England (19), and 72.0% in Germany (20). Insufficient prehospital EMS resources and the lack of awareness of stroke symptoms may partially explain the fewer EMS usage in China, which justifies specific stroke education strategies and proper EMS infrastructure planning for the targeted regions and population (21).

Consistent with previous studies (14, 22, 23), the EMS utilization differed between urban and rural regions, with significantly higher rates in urban areas. However, the prevalence, incidence, and mortality of stroke in China were significantly higher in rural than in urban areas (3). The disease burden of stroke consistently increased over the past 30 years, especially in the rural area. The imbalance in vascular risk factors, economic development, access to healthcare, and so on (22, 24, 25) may contribute to the urban-rural disparities in EMS utilization. Despite it, increasing year-on-year utilization of EMS over the past 4 years in both urban and rural areas has been observed in our study. Besides, the average annual growth rate in rural areas was nearly two times the rate in urban areas (12.6 vs. 6.4%, *P* < 0.001). It was worth noting that although rural residents were underutilizing EMS when compared to their urban counterparts, the significant decrements in urban-rural disparities reflect the positive effect of implementing a uniform stroke management model across the whole region.

We identified several factors associated with seeking EMS, including older age, more severe stroke, possessing atrial fibrillation or coronary artery disease, off-hour hospital arrival, and treatment in tertiary hospitals. The results could be used as a crucial basis for improving education strategies and EMS planning. The facilitators of EMS use in this study were similar to previous studies (6, 7, 12, 26, 27). A possible explanation is that patients with these characteristics are more likely to access EMS and realize the need for urgent stroke treatment. Older patients tend to be vulnerable and have more severe symptoms at the onset, which may prompt patients and bystanders to activate EMS as soon as symptoms manifest in case of more serious consequences (6, 26). Similarly, patients with higher NIHSS baseline scores presented with more severe and acute symptoms, which may prompt patients and bystanders to recognize stroke-related episodes and initiate EMS (6, 12). Consistent with another Chinese study that found that strong predictors of EMS use were cardiovascular disease (7), patients with atrial fibrillation or coronary artery disease in this study were more likely to activate EMS. The likely explanation is that these patients and their co-residents are well educated to use EMS, or even have used EMS multiple times. Patients with AIS onset outside of working hours may have difficulty using public transport or taxis after operating hours, raising the possibility that EMS will be selected. Treating in tertiary hospitals might indicate a higher level of income and health awareness and access to adequate EMS resources and in-hospital education, thus promoting recognition of stroke and decisions using EMS (28). In addition, consistent with what has been reported in other studies (6, 29, 30), we found that patients with prior stroke were less likely to use EMS, indicating potential missed opportunities to educate stroke or TIA patients on the need for EMS transportation at future symptom onset, and suggesting that in-hospital education should be strengthened due to the high recurrence rate of stroke. Previous studies have confirmed that patient knowledge regarding stroke is still lacking at the time of discharge (31, 32). It has also shown that awareness does not necessarily translate into appropriate actions to use the EMS immediately at stroke onset (32, 33). Therefore, not only should discharge education on stroke recognition be improved but timely EMS usage should also be vigorously advocated to reduce prehospital delays when suffering a relapse. Public education remains imminently reinforced, especially toward high-risk populations, such as individuals with advanced age, hypertension, prior stroke, and their families, to improve their ability to identify initial symptoms as stroke-related and recognize the urgency of contacting EMS.

In the stratification analysis, off-hour arrival and treatment in tertiary hospitals were more strongly correlated with EMS usage in urban areas than in rural areas. The underlying reasons for these characteristics cannot be determined right now. However, the various distributions of medical resources among regions and different levels of health literacy among populations may be an explanation for the disparities in the EMS utilization (34, 35). The association between sex and EMS usage was only observed in urban areas but not in rural areas, which may suggest the gap in awareness of seeking medical care related to socioeconomic status (36–38). The significant difference in EMS utilization between urban and rural areas suggests that special attention should be paid to rural areas to promote equal access to basic public health services and safeguard public welfare (39). Existing or newly developed stroke education programs need to expand their coverage and ensure efficient delivery of stroke knowledge to vulnerable high-risk people in rural areas (40, 41).

There are some limitations in the study. First, the research was carried out in Beijing with relatively rich medical resources, which could not represent other places lacking medical resources. As mentioned above, the EMS utilization rate in Beijing was significantly higher than that in other regions. The extrapolation of the results should be cautious. Second, the urban-rural division of Beijing based on administrative functions differed from the classical definition of urbanization. Nevertheless, previous studies have found significant differences between urban and rural residents in their use of chronic medical services (17), as well as the urban-rural differences in EMS use in our study. Third, based on the historical data from an administrative database, some factors such as economic status, health literacy, attitude to activate EMS, the performances of EMS on the therapy outcome and the long-term health should be evaluated when data are available.

## Conclusion

This study demonstrated an inadequate use of EMS among AIS patients in Beijing, especially in rural areas. Several factors were identified to be associated with EMS utilization including older age, more severe stroke, first stroke, possessing atrial fibrillation or coronary artery disease, off-hour hospital arrival, treating in tertiary hospitals, and living in urban communities. Enhanced education programs and EMS accessibility are still necessary, particularly for high-risk individuals and regions.

## Data availability statement

The raw data supporting the conclusions of this article will be made available by the authors, without undue reservation.

## Ethics statement

The studies involving human participants were reviewed and approved by the Ethics Committee of Peking University Health Science Center, Beijing, China. Written informed consent for participation was not required for this study in accordance with the national legislation and the institutional requirements.

## Author contributions

YWu and WenZ contributed to the study concept and had full access to all the data in the study. KD, YWu, and HC take responsibility for the integrity of the data, interpreted the findings, and drafted the article. KD, YWa, HL, and BC contributed to the data analysis. WeiZ, LG, GD, and TS interpreted the data. WenZ and YWu attests that all listed authors meet authorship criteria and that no others meeting the criteria have been omitted. All the authors contributed to the critical revision of the article for important intellectual content and approved the submitted version.

## Conflict of interest

The authors declare that the research was conducted in the absence of any commercial or financial relationships that could be construed as a potential conflict of interest.

## Publisher's note

All claims expressed in this article are solely those of the authors and do not necessarily represent those of their affiliated organizations, or those of the publisher, the editors and the reviewers. Any product that may be evaluated in this article, or claim that may be made by its manufacturer, is not guaranteed or endorsed by the publisher.
