# Supplementary material for: Emergency medical service utilization among acute ischemic stroke patients in Beijing: An observational study
Source: Front Neurol. 2022 Sep 6;13:969947. doi: 10.3389/fneur.2022.969947 (PMC9485477; doi:10.3389/fneur.2022.969947)
Supplement: Supplementary file 1 [file Data_Sheet_1.doc]

**Supplementary Table 1 74** **hospitals in the First Aid Treatment Map for Stroke (FATMS) in Beijing**

| **Urban areas** | **Rural areas** |
| --- | --- |
| Xicheng district | Tongzhou district |
| Xuanwu Hospital, Capital Medical University | Beijing Luhe Hospital, Capital Medical University |
| Beijing Friendship Hospital. Capital Medical University | Dongzhimen Hospital of Beijing University of Chinese Medicine (Tongzhou District) |
| Beijing Jiangong Hospital | Beijing Friendship Hospital affiliated to Capital Medical University (Tongzhou District) |
| Fuxing Hospital Affiliated to Capital Medical University | Daxing district |
| Peking University First Hospital | Beijing Daxing District People's Hospital |
| Peking University People's Hospital | Beijing Daxing District Integrated Traditional Chinese and Western Medicine Hospital |
| Chaoyang district | Beijing Renhe Hospital |
| Beijing Red Cross Emergency Rescue Center | South District of Beijing Tongren Hospital, Capital Medical University |
| Civil Aviation General Hospital | Changping district |
| Beijing Zhuyangliu Hospital | Beijing Tsinghua Changgung Hospital |
| Beijing Chaoyang Emergency Rescue Center | Beijing Changping District Hospital |
| China-japan Friendship Hospital | Peking University International Hospital |
| Beijing Huaxin Hospital | Beijing Changping District Integrated Traditional Chinese and Western Medicine Hospital |
| Beijing Chaoyang Hospital affiliated to Capital Medical University | Huilongguan Hospital of Beijing Jishuitan Hospital |
| Aviation General Hospital | Beijing Royal Hospital of Integrated Traditional Chinese and Western Medicine |
| Special Medical Center of PLA Strategic Support Force | Fangshan district |
| Beijing Anzhen Hospital, Capital Medical University | Beijing Fangshan District First Hospital |
| Beijing Chaoyang Integrated Traditional Chinese and Western Medicine Emergency Rescue Center | Liangxiang Hospital, Fangshan District. Beijing |
| Beijing Ditan Hospital. Capital Medical University | Beijing Yanhua Hospital |
| Wangjing Hospital, China Academy of Chinese Medical Sciences | Beijing Fangshan Hospital of Traditional Chinese Medicine |
| General Emergency Hospital (Coal General Hospital) | Shunyi district |
| Fengtai district | Beijing Shunyi District Hospital |
| Beijing Tiantan Hospital, Capital Medical University | Shunyi Airport Hospital, Beijing |
| Beijing Fengtai Right Anmen Hospital | Beijing Shunyi Hospital of Traditional Chinese Medicine |
| Beijing Electric Power Hospital | Mentougou district |
| Nanyuan Hospital, Fengtai District, Beijing | Beijing Beijing coal coke Group General Hospital |
| Dongfang Hospital of Beijing University of Chinese Medicine | Beijing Mentougou District Hospital |
| Beijing Fengtai Hospital | Pinggu district |
| Beijing Boai Hospital | Beijing Pinggu District Hospital |
| Tieying Hospital, Fengtai District, Beijing | Huairou district |
| Beijing Aerospace General Hospital | Beijing Huairou Hospital |
| Dongcheng district | Beijing Hospital of Traditional Chinese Medicine Huairou Hospital |
| Third Medical Center of PLA General Hospital (Armed Police) | Miyun district |
| Beijing Puren Hospital | Beijing Miyun District Hospital |
| Beijing Hospital of Traditional Chinese Medicine, Capital Medical University | Yanqing district |
| Beijing Hospital, Ministry of Health | Beijing Yanqing District Hospital |
| Peking Union Medical College Hospital, Chinese Academy of Medical Sciences |  |
| Beijing Tongren Hospital, Capital Medical University |  |
| Haidian district |  |
| Peking University Third Hospital |  |
| Space Center Hospital |  |
| Xiyuan Hospital, China Academy of Chinese Medical Sciences |  |
| Beijing Shijitan Hospital |  |
| Beijing Haidian Hospital |  |
| Beijing Geriatric Hospital |  |
| Beijing Zhongguancun Hospital |  |
| Jingdong Medical District, PLA General Hospital |  |
| Shijingshan district |  |
| Beijing Shijingshan Hospital |  |
| West Hospital of Beijing Chaoyang Hospital, Capital Medical University |  |
| Shougang Hospital of Peking University |  |
| Yuquan Hospital of Tsinghua University |  |

Supplementary Table 2 Characteristics of AIS patients by EMS use status

|  | Overall (N=24296) | EMS (N=11190) | Non-EMS (N=13106) | *P*-value |
| --- | --- | --- | --- | --- |
| Age, years, mean (sd) | 65.6 (12.8) | 67.9 (13.1) | 63.6 (12.2) | <0.001 |
| Male, n (%) | 16620 (68.4) | 7391 (66.1) | 9229 (70.4) | <0.001 |
| NIHSS, mean (sd) | 7.9 (6.9) | 10.8 (7.7) | 5.5 (5.1) | <0.001 |
| Comorbidity, n (%) |  |  |  |  |
| Hypertension | 14240 (58.6) | 6625 (59.2) | 7615 (58.1) | 0.085 |
| Diabetes mellitus | 6111 (25.2) | 2810 (25.1) | 3301 (25.2) | 0.905 |
| Prior Stroke | 6036 (24.8) | 2694 (24.1) | 3342 (25.5) | <0.001 |
| Coronary artery disease | 4505 (18.5) | 2430 (21.7) | 2075 (15.8) | <0.001 |
| Dyslipidemia | 2917 (12.0) | 1151 (10.3) | 1766 (13.5) | <0.001 |
| Atrial fibrillation | 2672 (11.0) | 1834 (16.4) | 838 (6.4) | <0.001 |
| PVD/CS/HF | 330 (13.6) | 167 (14.9) | 163 (12.4) | 0.107 |
| Off-hour arrival, n (%) | 9080 (37.4) | 4536 (40.5) | 4544 (34.7) | <0.001 |
| Weekend arrival, n (%) | 6706 (27.6) | 3162 (28.3) | 3544 (27.0) | 0.036 |
| Door-to-needle time≤60min, n/N* (%) | 12102/15601 (77.6) | 5035/6321 (79.7) | 7067/9280 (76.2) | <0.001 |
| Tertiary hospital, n (%) | 21544 (88.7) | 10277 (91.8) | 11267 (86.0) | <0.001 |

*N, total number of patients receiving intravenous thrombolytic therapy in the group.

**Supplementary Table 3** Factors associated with EMS utilization

|  | Total | | Urban | | Rural | |
| --- | --- | --- | --- | --- | --- | --- |
|  | OR (95%CI) | *P-value* | OR (95%CI) | *P-value* | OR (95%CI) | *P-value* |
| Age per 10 years | 1.20 (1.17, 1.23) | <0.001 | 1.18 (1.15, 1.22) | < 0.001 | 1.18 (1.13, 1.23) | < 0.001 |
| Sex |  |  |  |  |  |  |
| Female | ref |  | ref |  | ref |  |
| Male | 1.06 (1.00, 1.13) | 0.060 | 1.00 (0.92, 1.08) | 0.986 | 1.11 (1.01, 1.23) | 0.038 |
| NIHSS group |  |  |  |  |  |  |
| 0-5 | ref |  | ref |  | ref |  |
| 6-16 | 3.17 (2.98, 3.37) | <0.001 | 3.10 (2.85, 3.36) | < 0.001 | 3.27 (2.95, 3.62) | < 0.001 |
| >16 | 7.89 (7.01, 8.77) | <0.001 | 8.28 (7.14, 9.60) | < 0.001 | 8.00 (6.81, 9.39) | < 0.001 |
| Comorbidity, n (%) |  |  |  |  |  |  |
| Hypertension | 1.04 (0.98, 1.11) | 0.160 | 1.07 (0.98, 1.16) | 0.125 | 1.11 (1.01, 1.23) | 0.031 |
| Diabetes mellitus | 1.03 (0.96, 1.10) | 0.455 | 1.00 (0.92, 1.09) | 0.984 | 0.98 (0.87, 1.10) | 0.704 |
| Prior Stroke | 0.85 (0.79, 0.91) | <0.001 | 0.80 (0.73, 0.87) | < 0.001 | 0.89 (0.80, 1.00) | 0.055 |
| Coronary artery disease | 1.17 (1.08, 1.26) | <0.001 | 1.19 (1.07, 1.31) | < 0.001 | 1.18 (1.04, 1.34) | 0.013 |
| Atrial fibrillation | 1.60 (1.45, 1.77) | <0.001 | 1.53 (1.35, 1.74) | < 0.001 | 1.53 (1.29, 1.81) | < 0.001 |
| Dyslipidemia | 0.78 (0.71, 0.85) | <0.001 | 0.74 (0.66, 0.82) | < 0.001 | 0.77 (0.65, 0.91) | 0.002 |
| HF/CS/CVD | 0.94 (0.73, 1.20) | 0.618 | 1.30 (0.91, 1.84) | 0.146 | 0.69 (0.46, 1.03) | 0.070 |
| Off-hour arrival, n (%) | 1.30 (1.23,1.39) | < 0.001 | 1.37 (1.28, 1.49) | <0.001 | 1.15 (1.05, 1.27) | 0.003 |
| Weekend arrival, n (%) | 1.03 (0.97, 1.10) | 0.334 | 1.04 (0.96, 1.13) | 0.316 | 1.03 (0.92, 1.14) | 0.634 |
| Level of hospital |  |  |  |  |  |  |
| Secondary hospital | ref |  | ref |  | ref |  |
| Tertiary hospital | 1.76 (1.61, 1.93) | <0.001 | 2.03 (1.75, 2.35) | < 0.001 | 1.07 (0.95, 1.21) | 0.284 |

*OR and 95% CI were obtained by multivariable logistic regression models with the generalized estimating equations to account for within-hospital clustering. Variables included in multivariable models were age, sex, baseline NIHSS group, comorbidities, time of hospital arrival, level of receiving hospital, and calendar year.
